# Concussion burden and later‐life cardiovascular risk factors in former professional American‐style football players

**DOI:** 10.1002/acn3.52045

**Published:** 2024-05-29

**Authors:** Can Ozan Tan, Rachel Grashow, Roland Thorpe, Karen K. Miller, David M. Nathan, Saef Izzy, Farid Radmanesh, Jonathan H. Kim, Marc G. Weisskopf, Herman A. Taylor, Ross D. Zafonte, Aaron L. Baggish

**Affiliations:** ^1^ RAM Group, Department of Electrical Engineering, Mathematics, and Computer Science University of Twente the Netherlands; ^2^ Football Players Health Study at Harvard University Harvard Medical School Boston Massachusetts USA; ^3^ Department of Environmental Health Harvard T. H. Chan School of Public Health Boston Massachusetts USA; ^4^ Program of Research on Men's Health, Hopkins Center for Health Disparities Solutions Johns Hopkins Bloomberg School of Public Health Baltimore Maryland USA; ^5^ Department of Health Behavior and Society Johns Hopkins Bloomberg School of Public Health Baltimore Maryland USA; ^6^ Johns Hopkins Alzheimer's Disease Resource Center for Minority Aging Research Baltimore Maryland USA; ^7^ Neuroendocrine Unit Massachusetts General Hospital and Harvard Medical School Boston 02114 Massachusetts USA; ^8^ Diabetes Research Center Massachusetts General Hospital and Harvard Medical School Boston 02114 Massachusetts USA; ^9^ Department of Neurology, Divisions of Stroke, Cerebrovascular, and Critical Care Neurology Brigham and Women's Hospital, Harvard Medical School Boston Massachusetts USA; ^10^ Division of Neurocritical Care, Department of Neurology University of New Mexico Albuquerque New Mexico USA; ^11^ Emory Clinical Cardiovascular Research Institute Emory University School of Medicine Atlanta Georgia USA; ^12^ Cardiovascular Research Institute Morehouse School of Medicine Atlanta Georgia USA; ^13^ Department of Physical Medicine and Rehabilitation Spaulding Rehabilitation Hospital Charlestown Massachusetts USA; ^14^ Cardiovascular Performance Program Massachusetts General Hospital and Harvard Medical School Boston Massachusetts USA; ^15^ Department of Cardiology University of Lausanne Lausanne Switzerland; ^16^ Department of Sports Science University of Lausanne Lausanne Switzerland

## Abstract

**Objective:**

Mid‐life cardiovascular risk factors are associated with later cognitive decline. Whether repetitive head injury among professional athletes impacts cardiovascular risk is unknown. We investigated associations between concussion burden and postcareer hypertension, high cholesterol, and diabetes among former professional American‐style football (ASF) players.

**Methods:**

In a cross‐sectional study of 4080 professional ASF players conducted between January 2015 and March 2022, we used an mulitsymptom concussion symptom score (CSS) and the number of loss‐of‐consciousness (LOC) episodes as a single severe symptom to quantify football‐related concussion exposure. Primary outcomes were hypertension, dyslipidemia, and diabetes, defined by current or recommended prescription medication use.

**Results:**

The prevalence of hypertension, high cholesterol, and diabetes among former players (52 ± 14 years of age) was 37%, 34%, and 9%. Concussion burden was significantly associated with hypertension (lowest vs. highest CSS quartile, odds ratio (OR) = 1.99; 95%CI: 1.33–2.98; *p* < 0.01) and high cholesterol (lowest vs. moderate CSS, OR = 1.46, 95%CI, 1.11–1.91; *p* < 0.01), but not diabetes. In fully adjusted models, the prevalence of multiple CVD was associated with CSS. These results were driven by younger former players (≤ 40 year of age) in which the odds of hypertension were over three times higher in those in the highest CSS quartile (OR = 3.29, 95%CI: 1.39–7.61; *p* = 0.01). Results were similar for LOC analyses.

**Interpretation:**

Prior concussion burden is associated with postcareer atherogenic cardiovascular risk profiles among former professional American football players.

## Introduction

Mid‐life cardiovascular disease (CVD) is the leading noncommunicable cause of age‐related declines in brain health^1^ and represents the leading cause of death worldwide.^2^, ^3^, ^4^ While some of the CVD risk factors are not modifiable, early traumatic head injury is an increasingly recognized a risk factor for mid‐life adverse CVD outcomes in nonathlete patient populations.^5^, ^6^, ^7^, ^8^

American‐style football (ASF) players provide a unique opportunity to study such associations as they are exposed to repetitive traumatic brain injuries early in life.^9^ In fact, ASF participation has been associated with increased risk of traditional CVD risk factors including hypertension,^10^ hyperlipidemia,^11^ and diabetes mellitus,^12^, ^13^ during and after years of active play. Deliberate weight gain for performance advantage,^14^ acquired disorders of sleep,^15^ maladaptive concentric left ventricular hypertrophy,^16^ and habitual use of nonsteroidal anti‐inflammatory medications^17^ have been proposed as possible contributors to CVD among ASF players. Importantly, recent data demonstrate an association between intra‐career concussion burden and the isolated later‐life CVD risk factors in this population.^18^ Thus, ASF players serve as a unique population to delineate the contribution of early life repetitive concussion exposure to later‐life CVD risk.

We hypothesized that the burden of repetitive traumatic head injury during professional ASF participation would be associated with unfavorable later‐life CVD risk profiles. To address this hypothesis, we examined relationships between concussion symptom burden (a self‐reported metric of repetitive concussion), and number of loss‐of‐consciousness episodes (a previously used self‐reported metric of severe concussion^19^, ^20^, ^21^ that appears on other brain injury screens^22^, ^23^), and the prevalence of postcareer hypertension, hyperlipidemia, and diabetes mellitus among 4080 participants enrolled in the Football Players Health Study at Harvard University.^24^

## Methods

### Population

The Football Players Health Study (FPHS) at Harvard University^24^ enrolled former professional ASF players who contracted with the National Football League (NFL) after 1960 when plastic helmets were formally adopted.^25^ Using residential mail and email addresses obtained from the NFL Players Association, 15,015 former players were invited to participate in either a hardcopy or online questionnaire, out of which 4171 (27.8%) enrolled. The Beth Israel Deaconess Medical Center and the Harvard T. H. Chan School of Public Health institutional review boards approved this study, and all participants provided written or electronic informed consent prior to participation.

### Measures

Age was recorded based on the reported date of birth at the time of survey participation and represented as a continuous measure. All participants were male, and race was assigned as previously described,^26^ whereby participants chose the category that best described their race/ethnicity and were further classified into Black, White, Native Hawaiian/Pacific Islander/ Asian/American Indian/Alaskan Native, or “not reported.” We calculated body mass index (BMI) using self‐reported height and weight^27^ and categorized BMI as <25.0, 25.0–30.0, and >30.0 kg/m^2^. Smoking status was defined as never, former, current, and “not reported.”

Position was divided into linemen (defensive line, linebacker, and offensive line) and nonlinemen (defensive back, kicker/punter, quarterback, running back, tight end, wide receiver, and special teams only). Participants responded to survey questions on their debut year of professional play and the number of professional seasons of play. Final calendar year of play was used to determine years since professional play. At present, there is no objective method to assess repetitive head injury exposure. Accordingly, concussion symptom burden accrued during active play was investigated by asking “While playing or practicing football, did you experience a blow to the head, neck, or upper body followed by any of the following: dizziness, loss of consciousness, memory problems, disorientation, headaches, nausea, confusion, seizure, or feeling unsteady of your feet?” For each of these signs/symptoms, participants chose “none,” “once,” “2 to 5 times,” “6 to 10 times,” or “11 times.” As used previously^18^, ^28^, ^29^ estimates of symptom frequency were summed to create a concussion symptom score (CSS). Full CSS scale (0 to 130) was categorized into quartiles by dividing the range of possible scores (0–130) into four categories to reflect low, mild, moderate, and high levels of concussion symptom burden.

Study definitions of “hypertension or high blood pressure,” “high cholesterol,” and “diabetes or high blood sugar” were based on self‐reported medication history. Medication history was used to ascertain CVD risk factor status in an attempt to maximize the specificity of prevalence. Study participants were asked, “Has a medical provider ever recommended or prescribed medication for [condition]” and “Are you currently taking medication [for that condition]?” Participants who answered affirmatively to one or both of these disease‐specific medication questions were assigned that diagnosis. We utilized individual risk factors (hypertension, high cholesterol, or diabetes, coded as “0” or “1”) and total number of reported risk factors (each coded as 0–3) as outcome variables for statistical analysis.

### Statistical analysis

Descriptive variables were compared between quartiles of CSS using Kruskal–Wallis rank sum tests for continuous variables and Fisher's test (with Monte Carlo simulation to compute p‐values) for categorical variables.^30^ We utilized two generalized linear models with a binomial link function to assess the relation of demographic and football‐related variables to CVD risk factors. All statistical models included age, race, BMI, smoking status, linemen status, number of professional seasons, years since active play, and CSS categories as exposure (i.e., independent) variables. The first model was used to estimate odds ratio of one or more CVD risk factors (compared to “none”) as an outcome variable. The second model was used to estimate the odds ratio of individual CVD risk factors (hypertension, hyperlipidemia, and diabetes mellitus) separately.

We performed three a priori specified sensitivity analyses. First, we used an ordered logistic regression model to examine if using each level of CVD factors (none, either of the three, two or more) reflects the contrasts of those with two or more CVD risk factors. Second, we examined the relationship between CSS and CVD risk factor prevalence in the subgroup of men ≤40 years of age. We undertook this analysis to simultaneously minimize the impact of advancing age and thereby to isolate the impact of ASF participation on CVD risk factor prevalence. Age 40 was chosen as this represents the recommended age to begin comprehensive CVD risk factor assessment by contemporary clinical guidelines.^31^ Third, we used self‐reported number of loss‐of‐consciousness (LOC) events, an established surrogate in sport and military veteran cohorts^19^, ^20^, ^21^ for cumulative head injury burden, to reduce the potential influence of recall bias. All statistical analyses were conducted using R Language for Statistical Computing,^27^ and relationships with a p‐value of 0.05 or lower were considered statistically significance.

## Results

The cohort included in the final analysis, after the removal of 91 participants who did not fully report their concussion‐related symptom burden, included 4080 men (52 ± 14 years, BMI of 31 ± 5 kg/m^2^; Table 1). Former ASF players, among whom 1586 (39%) identified as Black, reported 7 ± 4 years of NFL play during which 1386 (34.0%) played at a linemen field position. Prevalence estimates (95% CI) for the three principal CVD risk factors, hypertension, high cholesterol, and diabetes, were 37.0 (35.6–38.5)%, 33.4 (31.9–34.8)%, and 8.8 (8.0–9.8)%, respectively.

**Table 1:** Cohort characteristics.

Characteristic	Overall, *N* = 4080^a^	Very low CSS <33, *N* = 2604^a^	Mild CSS 33–65, *N* = 965^a^	Moderate CSS 65–98, *N* = 369^a^	High CSS >98, *N* = 142^a^	*p*‐value^b^
Concussion symptom score (CSS)	30.7 (27.1)	14.1 (9.3)	45.6 (9.1)	78.7 (9.0)	109.5 (9.1)	<0.001
Number of loss‐of‐consciousness episodes (LOC)						<0.001
0	1986 (49.4%)	1554 (60.7%)	323 (33.7%)	95 (26.2%)	14 (9.9%)	
1	883 (21.9%)	620 (24.2%)	207 (21.6%)	49 (13.5%)	7 (5.0%)	
4	930 (23.1%)	382 (14.9%)	368 (38.4%)	147 (40.5%)	33 (23.4%)	
8	131 (3.3%)	4 (0.2%)	56 (5.8%)	57 (15.7%)	14 (9.9%)	
13	93 (2.3%)	0 (0.0%)	5 (0.5%)	15 (4.1%)	73 (51.8%)	
Missing						
Age	51.6 (14.4)	52.8 (14.9)	49.8 (13.2)	49.4 (13.1)	49.2 (12.3)	<0.001
Race						<0.001
White	2322 (57.6%)	1521 (59.3%)	544 (56.8%)	193 (52.7%)	64 (45.1%)	
Black	1586 (39.3%)	975 (38.0%)	392 (41.0%)	152 (41.5%)	67 (47.2%)	
Native Hawaiian, Pacific Islander, Asian, American Indian, and Alaskan Native	124 (3.1%)	71 (2.8%)	21 (2.2%)	21 (5.7%)	11 (7.7%)	
Missing	48	37	8	3	0	
BMI	31.3 (5.0)	31.0 (5.0)	31.6 (5.0)	32.2 (5.1)	32.8 (5.4)	<0.001
Missing	28	19	7	1	1	
Smoking						0.6
Never smoked	3396 (83.9%)	2186 (84.7%)	787 (82.2%)	305 (83.1%)	118 (84.3%)	
Current smoker	522 (12.9%)	322 (12.5%)	132 (13.8%)	49 (13.4%)	19 (13.6%)	
Former smoker	128 (3.2%)	74 (2.9%)	38 (4.0%)	13 (3.5%)	3 (2.1%)	
Missing	34	22	8	2	2	
Linemen	1386 (34.0%)	861 (33.1%)	339 (35.1%)	138 (37.4%)	48 (33.8%)	0.3
Number of professional seasons	6.7 (3.9)	6.5 (3.9)	6.7 (3.8)	7.1 (3.6)	7.3 (3.8)	<0.001
Missing	2	2	0	0	0	
Debut season of play						<0.001
1950–1959	780 (19.2%)	464 (17.9%)	204 (21.3%)	81 (22.2%)	31 (21.8%)	
1960–1969	196 (4.8%)	139 (5.4%)	41 (4.3%)	12 (3.3%)	4 (2.8%)	
1970–1979	83 (2.0%)	69 (2.7%)	9 (0.9%)	3 (0.8%)	2 (1.4%)	
1980–1989	550 (13.6%)	426 (16.5%)	85 (8.9%)	30 (8.2%)	9 (6.3%)	
1990–1999	799 (19.7%)	539 (20.8%)	168 (17.5%)	67 (18.4%)	25 (17.6%)	
2000–2009	928 (22.9%)	526 (20.3%)	262 (27.3%)	99 (27.1%)	41 (28.9%)	
2010–2022	719 (17.7%)	425 (16.4%)	191 (19.9%)	73 (20.0%)	30 (21.1%)	
Missing	25	16	5	4	0	
Years since NFL play	23.4 (14.3)	24.7 (14.9)	21.5 (12.9)	20.8 (12.8)	20.3 (12.4)	<0.001
Missing	6	5	1	0	0	
Diabetes	356 (8.9%)	231 (9.1%)	74 (7.8%)	33 (9.3%)	18 (12.9%)	0.2
Missing	85	52	18	13	2	
Hypertension	1507 (37.2%)	945 (36.6%)	349 (36.4%)	143 (39.1%)	70 (49.6%)	0.014
Missing	29	19	6	3	1	
High cholesterol	1350 (33.7%)	841 (32.8%)	315 (33.3%)	140 (38.4%)	54 (39.1%)	0.10
Missing	69	42	19	4	4	
Maximum ever BMI						0.003
BMI <25	73 (1.8%)	54 (2.1%)	13 (1.4%)	5 (1.4%)	1 (0.7%)	
BMI 25–30	1182 (29.2%)	810 (31.4%)	253 (26.3%)	90 (24.5%)	29 (20.6%)	
BMI >30	2794 (69.0%)	1715 (66.5%)	696 (72.3%)	272 (74.1%)	111 (78.7%)	
Missing	31	25	3	2	1	
Obesity	2157 (53.2%)	1310 (50.7%)	532 (55.5%)	222 (60.3%)	93 (66.0%)	<0.001
Missing	28	19	7	1	1	

^a^ Mean (SD); *n* (%).

^b^ Kruskal–Wallis rank sum test; Fisher's exact test for count data with simulated *p*‐value (based on 2000 replicates).

**Figure 1:**
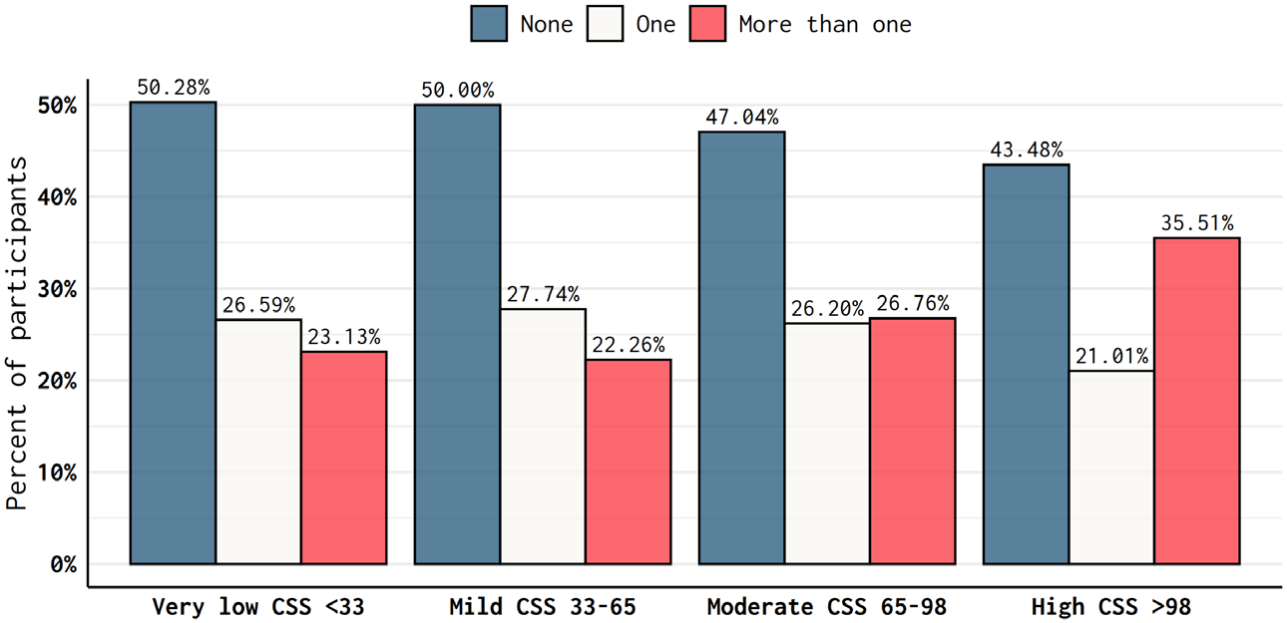
Number of cardiovascular risk factors (hypertension, high cholesterol, and/or diabetes) in participants who reported very low, mild, moderate, and high concussion symptom scores.

The average CSS was 31 ± 27 (range 0 to 130). In unadjusted analyses, the prevalence of former players who reported no CVD risk factors decreased across the CSS quartiles. Conversely, the proportion of players with multiple CV risk factors increased with increasing concussion symptom quartile (Fig. 1). More than one‐third of former players with more than one cardiovascular risk factor were in the highest CSS quartile. Pairwise comparisons of the three cardiovascular outcomes did not provide evidence of collinearity (all *p* < 0.001; data not show).

**Figure 2:**
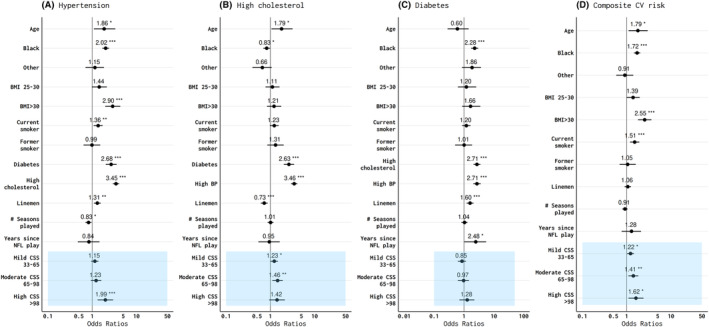
Adjusted odds ratios of self‐reported cardiovascular risk factors including (A) hypertension; (B) high cholesterol; (C) diabetes; and (D) composite cardiovascular (CV) risk (including hypertension, high cholesterol and/or diabetes) among former professional American‐style football players. Models included age, race, body mass index (BMI), smoking status, linemen status, cardiovascular risk factors, number of professional seasons, era of debut season (not shown), years since professional play, and concussion symptom score. Grades of concussion score include mild (33–64), moderate (65–97), and high (>98) compared to the low (0–33) reference group. White race, BMI <25.0, nonsmokers, nondiabetics, nonlinemen, those with a debut season prior to 1960, and low concussion symptom burden <33 served as reference groups for age, race, BMI, smoking, position, debut seasons, and concussion symptom quartile respectively. *p < 0.05; **p < 0.01, ***p < 0.001.

Multivariable models that used hypertension, high cholesterol, diabetes, and two or more CVD risk factors as the dependent variable are shown in (Fig. 2). Significant variables in the hypertension model included age, race, smoking, BMI >30 kg/m^2^, linemen status, diabetes, high cholesterol, and CSS quartile. Notably, CSS quartile showed an independent and direct relationship with prevalent hypertension. In contrast, longer careers, as measured by “season of professional play,” showed a significantly protective effect against hypertension (Fig. 2A).

**Figure 3:**
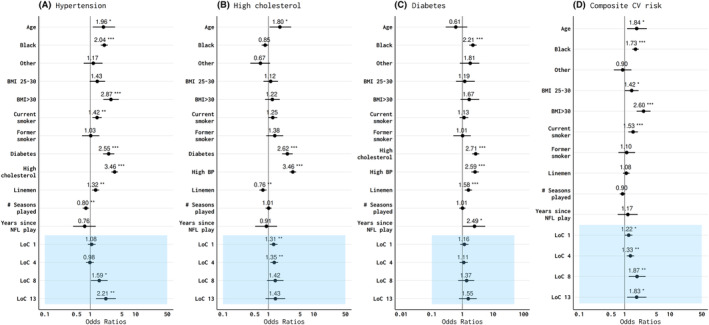
Adjusted odds ratios of self‐reported cardiovascular risk factors including (A) hypertension; (B) high cholesterol; (C) diabetes; and (D) composite cardiovascular (CV) risk among former professional American‐style football players from a model that included age, race, body mass index (BMI), smoking status, linemen status, cardiovascular risk factors, number of professional seasons, era of debut season (not shown), years since professional play, and number of loss‐of‐consciousness (LOC) episodes. White race, BMI <25.0, nonsmokers, nondiabetics, nonlinemen, those with a debut season prior to 1960, and zero LOC episodes served as reference groups for age, race, BMI, smoking, position, debut seasons, and number of LOC episodes respectively. *p < 0.05; **p < 0.01, ***p < 0.001.

Factors that were significant associated with high cholesterol included age, diabetes, and hypertension. In contrast, Black race and linemen field position status demonstrated a protective effect against high cholesterol (Fig. 2B). Mild and moderate concussion symptom burden were significantly associated with the odds of high cholesterol, while those in the highest concussion symptom score showed a positive but nonsignificant association with high cholesterol. Significant contributing factors for diabetes included Black race, linemen field position status, hypertension, and high cholesterol (Fig. 2C). No significant associations were observed between concussion symptom score and diabetes. When hypertension, high cholesterol, and diabetes were combined into a single composite score, age, race, BMI >30 kg/m^2^, and smoking were associated with the presence of multiple cardiovascular risk factor (Fig. 2D). We also observed a significant and direct relationship between the presence >1 CVD risk factor and CSS, such that men in the highest CSS quartile were the most likely to have more than one CVD risk factor. To study more severe head injury exposure that is less susceptible to recall bias, we used the number of loss‐of‐consciousness (LOC) episodes during active play in analyses and found similar results (Fig. 3).

**Figure 4:**
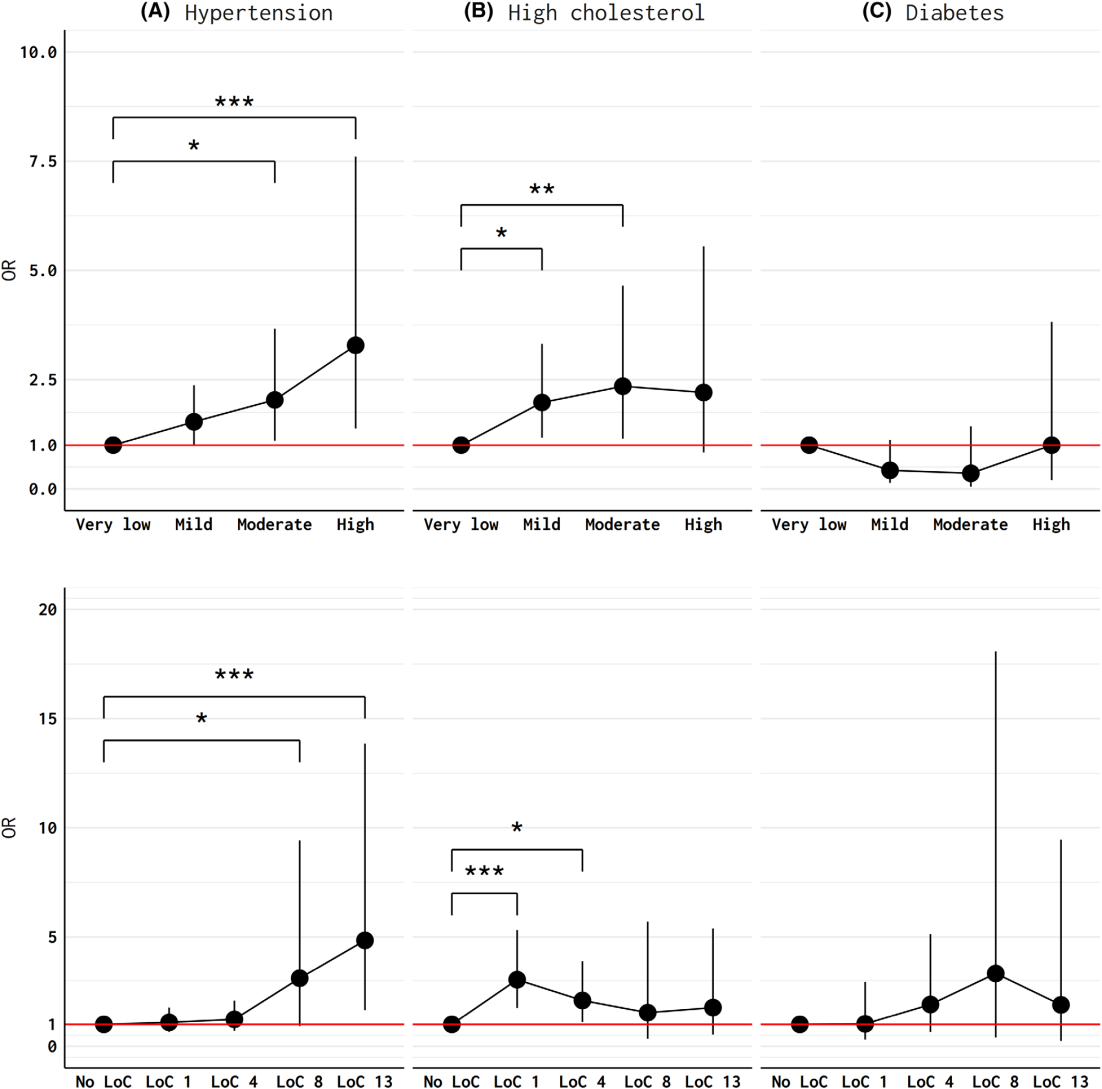
Adjusted odds ratios of self‐reported cardiovascular risk factors including (A) hypertension; (B) high cholesterol; and (C) diabetes among former professional American‐style football players ≤40 years of age. Top panel displays results from models included age, race, body mass index (BMI), smoking status, linemen status, cardiovascular risk factors, number of professional seasons, era of debut season, years since professional play, and concussion symptom score. Grades of concussion symptom score include mild (33–64), moderate (65–97), and high (>98) compared to the low (0–33) reference group. Lower panes display results from models that included age, race, body mass index (BMI), smoking status, linemen status, cardiovascular risk factors, number of seasons played, era of debut season, years since professional play, and number of loss‐of‐consciousness (LOC) episodes. White race, BMI <25.0, nonsmokers, nondiabetics, nonlinemen, those with a debut season prior to 1960, low concussion symptom burden <33, and no LOC episodes served as reference groups for age, race, BMI, smoking, position, debut seasons, concussion symptom quartile, and LOC respectively. Red line denotes the null odds ratio value.

Using each level of CVD factors (none, either of the three, two or more) instead of two or more CVD risk factors did not change our results (Fig. S1). Acknowledging the expected association between advancing age and the three CVD risk factors of interest, we performed a subgroup analysis confined to men ≤40 years of age (*n* = 1155). Among this cohort of relatively younger players, we found a statistically significant association between CSS quartile and hypertension, with those in the highest CSS quartile demonstrating an odds ratio of >3.0 compared to referents in the lowest CSS quartile (Fig. 4, top panels). Similar results were seen in LOC analyses (Fig. 4, bottom panels). In older players, we found significantly increased odds of hypertension in the highest CSS only (Table S1). The odds ratio of hypertension in the younger group (comparison group) with high CSS was more than double the odds ratio estimate for the older players (reference) in the highest CSS quartile, despite the high variability due to the relatively small number of participants (*n* = 126) in the highest CSS quartile. Results were similar for high cholesterol, but there were no significant associations between CSS and odds of prevalent diabetes in either age group.

## Discussion

This study was designed to examine the relationship between repetitive head injury accrued during participation in professional ASF and later‐life CVD risk factors. We performed these analyses to provide further insight into the pathogenesis of CVD, the leading cause of death among former ASF athletes.^9^ This study builds upon previous work which looked exclusively at hypertension without additional adjustments for other cardiovascular comorbidities.^18^ In the present study, we found that concussion symptom score (CSS), a surrogate metric for cumulative intra‐career head injury, was associated with CVD risk factor prevalence. This observation remained significant after adjustment for well‐established determinants of CVD risk factors including age, race, smoking, and BMI. These results were notable for hypertension and dyslipidemia, but not diabetes which occurred at a comparatively lower rate and independently of head injury exposure. In alignment with prior studies of non‐ASF populations,^5^, ^6^, ^7^ our data suggest that repetitive head injury increases the prevalence of CVD risk factors and may represent a previously unrecognized link between ASF participation and CVD.

Prior work has established relationships between ASF participation and the development of CVD risk factors. Longitudinal studies of collegiate ASF players have demonstrated the development of hypertension,^10^ vascular stiffness,^16^, ^32^ and concentric left ventricular hypertrophy.^7^, ^33^ Cross‐sectional data characterizing professional ASF athletes during active NFL participation documented an increased prevalence of hypertension compared with the general population.^34^ Proposed explanations for these observations include intra‐career weight gain,^34^ Black race,^35^ participation at the linemen field position,^35^ and use of nonsteroidal anti‐inflammatory agents.^30^ However, disentangling social determinants of health and football exposures may be difficult due to historical race‐based patterns of field position assignment.^36^ To date, the impact of head injury, a factor that has been shown to confer CVD risk in nonathlete populations, has not been similarly explored among competitive athletes in contact sports with high rates of head trauma. We recently reported an independent association between concussion symptom burden and hypertension,^18^ and now provide a more comprehensive assessment of the relationship between concussion burden in ASF players and later‐life CVD risk profiles.

The pathophysiologic link between antecedent head injury and CVD factors represents an area of active investigation. While the mechanisms delineating this phenomenon remain incompletely understood, several biologically plausible explanations including neuroinflammation,^37^ disruption of the bidirectional brain–gut axis,^38^ hypopituitarism,^39^ and autonomic nervous system dysfunction have been proposed.^40^, ^41^ Targeted investigation of how head injuries lead to the development CVD risk factors and subsequent adverse CVD outcomes represents an area of important area for future work. Unexpectedly, this study also found that BMI was not associated with hyperlipidemia and diabetes in multivariable models. Future studies should explore the extent to which BMI is a useful representation of body habitus in former players, and what other contributors may be driving late‐life health.

There are direct clinical implications of our findings. Consensus sports cardiology recommendations endorse screening young competitive athletes for genetic and congenital forms of CVD,^42^ and general clinical guidelines suggest comprehensive CVD risk factor assessment among all people age 40 and above.^31^ At present, screening recommendations for relatively youthful former competitive athletes below 40 years of age, a population demonstrated by our findings to harbor a significant burden of traditional CVD risk factors, are lacking. Our data suggest a role for accelerated CVD risk factor assessment among former professional ASF athletes. This strategy, tailored on an individual patient basis by obtaining a careful concussion history, may provide otherwise unrecognized opportunities for lifestyle and pharmacologic CVD risk reduction. The impact of this proposed approach on CVD outcomes and its applicability to other populations at risk for early life injury represent important areas of future work.

These findings may seem contradictory when juxtaposed with protective cardiovascular effects seen in elite athletes from other sports and other studies of ASF players.^35^, ^43^, ^44^ However, head and orthopedic injuries accrued during professional play may differ by magnitude, direction, and frequency across ASF, rugby, and European‐style football. Helmet use and type along with distinct profiles of cardioprotective aerobic activity may also produce variability in outcomes across sports. Finally, the reduced mortality seen in previous papers^35^ on professional ASF players that used general population comparisons may be due to healthy worker bias,^45^ where the factors that lead to an individual successfully competing for an NFL contract led them to healthier than the general population.

We acknowledge several limitations of this work. First, we relied on self‐reported symptoms to calculate the CSS as there are no superior self‐reported alternatives to retrospectively assess concussion burden. Given that our endpoints are cardiovascular and cardiometabolic (for which there is little to no public association with concussion), we would not expect to see the recall bias noted in other studies for participants who reported adverse cognitive and neuropsychological endpoints.^46^ Furthermore, in contrast to number of diagnosed concussions, the use of concussion symptom reporting reduces the impact of changing definitions of concussion over time and players' tendency to underreport injuries to coaches and team physicians.^47^, ^48^ Notably, our key findings were similar when we used number of loss‐of‐consciousness episodes, a head injury exposure measure largely regarded as being less susceptible to recall bias.^49^, ^50^, ^51^ Second, the CSS collapses mild, moderate, and severe concussion symptoms. While we separately analyzed one of the most severe symptoms (number of LOC episodes), these analyses cannot draw conclusions related to the long term cardiovascular and cardiometabolic effects of mild or moderate head symptoms resulting from a head injury. Third, the prevalence of traditional CVD risk factors was assessed by medication use profiles, rather than self‐reported diagnoses. We chose this approach to maximize the specificity of CVD risk factors ascertainment but acknowledge the inherent sensitivity trade‐off which may have led to incomplete capture of CVD risk factor prevalence. Third, we acknowledge the possibility of selection bias as our cohort was derived from an ongoing longitudinal cohort study with incomplete capture of the full source population of former professional ASF players. However, a study comparing the FPHS cohort to publicly available professional football data shows that the FPHS was representative across age, height, weight, and position.^24^ Fourth, we did not explore differences by survey administration format (pen and paper versus online), although age‐related preferences for survey format likely would be partially addressed through the inclusion of age as a covariate. Fifth, the finding that longer professional careers were protective against reporting CVD risk factor may reflect survivorship bias, a common occupational epidemiological finding such that those with the shortest and longest careers are often least likely to report late‐life conditions^45^ (e.g., sleep apnea or arthritis). Survivorship bias stems from the phenomenon whereby those with unmeasured protective genetic, biopsychosocial, or individual advantages enjoy longer careers with fewer adverse outcomes. This type of bias may not have been apparent in previous smaller cohorts have recruited symptomatic players that selectively reported a moderate number of years of professional play.

It should be additionally noted that these results can only be applied to males, and that additional research is needed to determine the extent to which these associations are present for study cohorts that include women. Furthermore, biased results would only occur if participation in our study was associated with selection both for concussion symptom reporting and CVD which is unlikely given our recruitment protocol and the size of our cohort. Finally, our cross‐sectional study design does not permit a definitive determination of causal mechanisms linking early life head injury to later‐life CVD risk. This represents an area of important future work which will require longitudinal repeated measures study designs and careful phenotyping.

In conclusion, our data suggest that repetitive head injury during professional ASF participation represents an independent determinant of later‐life CVD risk factor profiles. This finding, in turn, highlights the need to perform careful CVD risk factor assessment among physically active populations that are known to be susceptible, through recreation or occupation, to concussions. Future work will be required to define the relationship between early life concussion and the incidence of later‐life adverse cardiovascular events and mortality.

## Author Contributions

Conception and design of the study: COT, RG, RT, KKM, DN, RDZ, MGW, and ALB. Acquisition and analysis of data: COT, RG, RDZ, and ALB. Drafting a significant portion of the manuscript: COT, RG, RT, KKM, DN, SI, FR, JK, MGW, HT, RDZ, and ALB.

## Conflict of Interest

Dr. Zafonte reported receiving royalties from Springer/Demos publishing for serving as coeditor of the text Brain Injury Medicine, serving on the scientific advisory board of Myomo Inc., and onecare.ai Inc, evaluating patients in the Massachusetts General Hospital Brain and Body–TRUST Program, which is funded by the NFL Players Association; and receiving grants from the NIH; Dr. Baggish has received funding from the National Institute of Health/National Heart, Lung, and Blood Institute, the National Football Players Association, and the American Heart Association, and receives compensation for his role as team cardiologist from the US Olympic Committee/US Olympic Training Centers, US Soccer, US Rowing, the New England Patriots, the Boston Bruins, the New England Revolution, and Harvard University; Dr. Taylor reported receiving grants from the NFL Players Association outside the submitted work, and grants from the NIH; Dr. Weisskopf reported receiving grants from the NFL Players Association, and the NIH during the conduct of the study; Dr. Kim is the team cardiologist for the Atlanta Falcons; Dr. Grashow received grant funding from the NFL Players Association. No other disclosures were reported.

## Supporting information


Appendix S1.

## Data Availability

Due to the high profile nature of the participant population, these data are not available for external investigators.
