# Supplementary material for: Concussion burden and later‐life cardiovascular risk factors in former professional American‐style football players
Source: Ann Clin Transl Neurol. 2024 May 29;11(6):1604–14. doi: 10.1002/acn3.52045 (PMC11187967; doi:10.1002/acn3.52045)
Supplement: Supplementary file 1 — Appendix S1. [file ACN3-11-1604-s001.docx]

**Figure S1:** Odds ratio of self-reported composite cardiovascular risk factors when categorized as ordinal values (none, one, or more than one)  among former professional American-style football players. Top panel shows the hyperparameter alpha estimated by the ordinal regression (one risk factor vs none, more than one vs one). Bottom panel shows the adjusted odds ratios estimated using the ordinal regression model. The model included age, race, body mass index (BMI), smoking status, linemen status, cardiovascular risk factors, number of seasons played, era of debut season (not shown), years since professional play, and concussion symptom score. Grades of concussion score include mild (33-64), moderate (65-97), and high (>98) compared to the low (0-33) reference group. White race, BMI <25.0, non-smokers, non-diabetics, non-linemen, those with a debut season prior to 1960, and low concussion symptom burden <33 served as reference groups for age, race, BMI, smoking, position, debut seasons, and concussion symptom quartile respectively. Note that the adjusted odds ratios estimated with this model (bottom panel) are qualitatively not different from thos estimated using the main logistic regression model (none vs one or more; Figure 2D) *p < 0.05; **p < 0.01, ***p < 0.001.


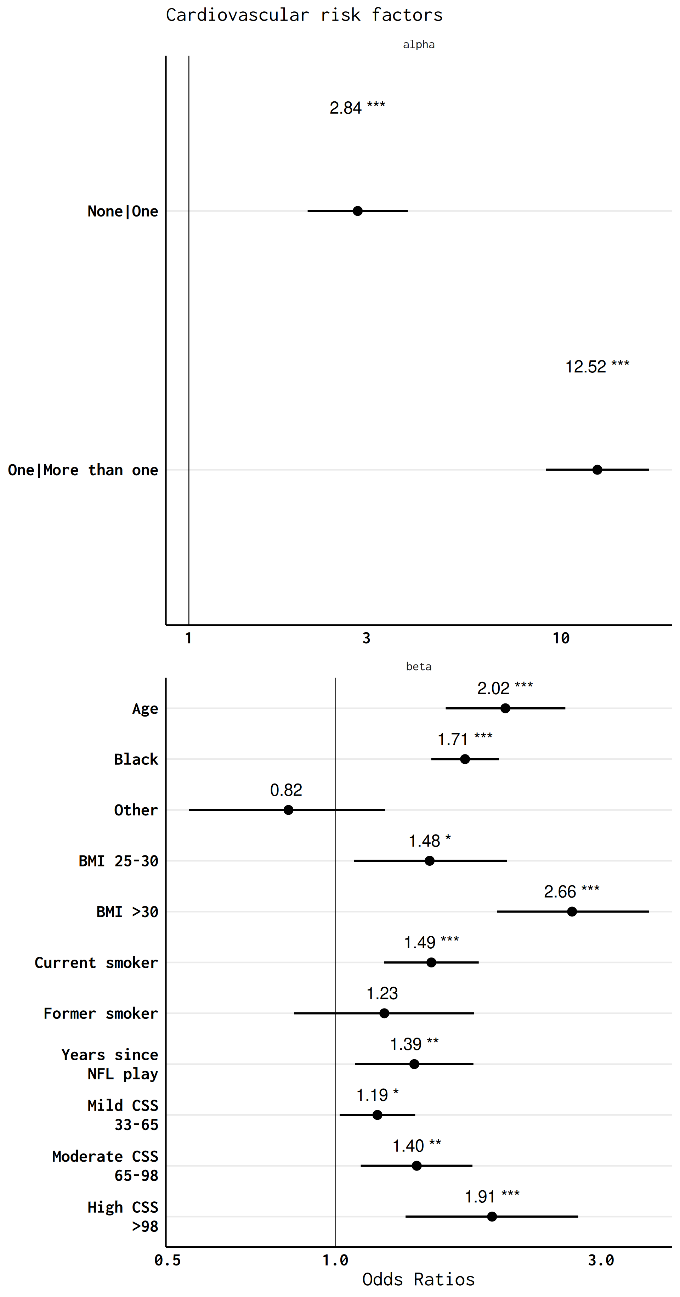


**Table S1:** Adjusted odds ratios of self-reported cardiovascular risk factors including (A) hypertension; (B) high cholesterol; (C) diabetes; and (D) composite cardiovascular (CV) risk among former professional American-style football players stratified at age 40. Models included age, race, body mass index (BMI), smoking status, linemen status, cardiovascular risk factors, number of professional seasons, era of debut season (not shown), years since professional play, and concussion symptom score. Grades of concussion score include mild (33-64), moderate (65-97), and high (>98) compared to the low (0-33) reference group. White race, BMI <25.0, non-smokers, non-diabetics, non-linemen, those with a debut season prior to 1960, and low concussion symptom burden <33 served as reference groups for age, race, BMI, smoking, position, debut seasons, and concussion symptom quartile respectively.

|  | **Age** | |
| --- | --- | --- |
| **Concussion symptom score** | **<=40 years of age**  **N = 1155** | **>40 years of age**  **N = 3016** |
| *Model 1: Hypertension* |  | |
| Very low (CSS <33) | Reference | |
| Mild (CSS 33-65) | 1.54 (0.99 - 2.38), p = 0.06 | 1.06 (0.86 - 1.30), p = 0.61 |
| Moderate (CSS 65-98) | **2.04 (1.10 - 3.66), p = 0.02** | 1.05 (0.77 - 1.42), p = 0.78 |
| High (CSS >98) | **3.29 (1.39 - 7.61), p = 0.01** | **1.64 (1.03 - 2.62), p = 0.04** |
| *Model 2: High cholesterol* |  | |
| Very low (CSS <33) | Reference | |
| Mild (CSS 33-65) | **1.99 (1.18 - 3.34), p = 0.01** | 1.13 (0.92 - 1.39), p = 0.23 |
| Moderate (CSS 65-98) | **2.35 (1.15 - 4.66), p = 0.02** | 1.33 (0.99 - 1.78), p = 0.06 |
| High (CSS >98) | 2.22 (0.84 - 5.57), p = 0.10 | 1.19 (0.75 - 1.86), p = 0.46 |
| *Model 3: Diabetes* |  | |
| Very low (CSS <33) | Reference | |
| Mild (CSS 33-65) | 0.44 (0.14 - 1.17), p = 0.12 | 0.90 (0.64 - 1.23), p = 0.51 |
| Moderate (CSS 65-98) | 0.37 (0.05 - 1.48), p = 0.22 | 1.05 (0.66 - 1.61), p = 0.84 |
| High (CSS >98) | 1.03 (0.20 - 3.95), p = 0.96 | 1.25 (0.64 - 2.28), p = 0.49 |
